# DNMT1 regulates the timing of DNA methylation by DNMT3 in an enzymatic activity-dependent manner in mouse embryonic stem cells

**DOI:** 10.1371/journal.pone.0262277

**Published:** 2022-01-05

**Authors:** Takamasa Ito, Musashi Kubiura-Ichimaru, Yuri Murakami, Aaron B. Bogutz, Louis Lefebvre, Isao Suetake, Shoji Tajima, Masako Tada

**Affiliations:** 1 Stem Cells & Reprogramming Laboratory, Department of Biology, Faculty of Science, Toho University, Funabashi, Chiba, Japan; 2 Department of Medical Genetics, Life Sciences Institute, University of British Columbia, Vancouver, British Columbia, Canada; 3 Department of Nutritional Sciences, Graduate School of Nutritional Sciences, Nakamura Gakuen University, Fukuoka, Japan; 4 Laboratory of Epigenetics, Institute for Protein Research, Osaka University, Suita, Osaka, Japan; Universität Stuttgart, GERMANY

## Abstract

DNA methylation (DNAme; 5-methylcytosine, 5mC) plays an essential role in mammalian development, and the 5mC profile is regulated by a balance of opposing enzymatic activities: DNA methyltransferases (DNMTs) and Ten-eleven translocation dioxygenases (TETs). In mouse embryonic stem cells (ESCs), *de novo* DNAme by DNMT3 family enzymes, demethylation by the TET-mediated conversion of 5mC to 5-hydroxymethylation (5hmC), and maintenance of the remaining DNAme by DNMT1 are actively repeated throughout cell cycles, dynamically forming a constant 5mC profile. Nevertheless, the detailed mechanism and physiological significance of this active cyclic DNA modification in mouse ESCs remain unclear. Here by visualizing the localization of DNA modifications on metaphase chromosomes and comparing whole-genome methylation profiles before and after the mid-S phase in ESCs lacking *Dnmt1* (1KO ESCs), we demonstrated that in 1KO ESCs, DNMT3-mediated remethylation was interrupted during and after DNA replication. This results in a marked asymmetry in the distribution of 5hmC between sister chromatids at mitosis, with one chromatid being almost no 5hmC. When introduced in 1KO ESCs, the catalytically inactive form of DNMT1 (DNMT1^CI^) induced an increase in DNAme in pericentric heterochromatin and the DNAme-independent repression of IAPEz, a retrotransposon family, in 1KO ESCs. However, DNMT1^CI^ could not restore the ability of DNMT3 to methylate unmodified dsDNA *de novo* in S phase in 1KO ESCs. Furthermore, during *in vitro* differentiation into epiblasts, 1KO ESCs expressing DNMT1^CI^ showed an even stronger tendency to differentiate into the primitive endoderm than 1KO ESCs and were readily reprogrammed into the primitive streak via an epiblast-like cell state, reconfirming the importance of DNMT1 enzymatic activity at the onset of epiblast differentiation. These results indicate a novel function of DNMT1, in which DNMT1 actively regulates the timing and genomic targets of *de novo* methylation by DNMT3 in an enzymatic activity-dependent and independent manner, respectively.

## Introduction

DNA methylation (DNAme) is an essential epigenetic mark in mammalian development, and it regulates chromatin structure and gene expression through interaction with other proteins [1–3]. In mice, the *de novo* DNA methyltransferases DNMT3A/3B/3C (DNMT3s) convert unmodified cytosine to 5-methylcytosine (5mC), establishing cell-type-specific DNAme profiles [4, 5]. In addition, DNMT3L, which has no enzymatic activity, functions as an accessory protein for other DNMT3 enzymes [6], and plays an essential role in acquiring genomic imprints during late germ cell development [7, 8]. An accessory function that enhances *de novo* DNMT activity is also found in DNMT3B. Both the catalytically inactive (CI) forms DNMT3B3, an isoform lacking key exons, and DNMT3B^CI^, an artificial amino acid substitution, replace DNMT3B function almost completely in mouse embryonic stem cells (ESCs) and mice, respectively [9, 10]. DNMT1, a maintenance methyltransferase, copies methylation patterns on the nascent DNA strand during the S phase and partly during the G2 phase. Its deficiency leads to embryonic lethality with abnormal expression patterns, including increased expression of transposable elements [11–13]. DNMT1 also interacts with many proteins required for DNA binding at replicating regions, hemimethylated regions, and heterochromatin. For example, the ubiquitin E3 ligase UHRF1/Np95 (ubiquitin-like with PHD and RING finger domains) that ubiquitinates both PAF15 and histone H3 in the region that is rich in lysine 9-methylated histone H3 (H3K9me2/3) is an essential protein that directs the recruitment of DNMT1 to replicating regions and heterochromatin [14–18]. The de novo DNAme activity of DNMT1 has been reported in an in-vitro study [19], and the UHRF1-coupled de novo DNAme activity of DNMT1 has been documented in retrotransposon repeats in mice [20, 21]. Thus, the cooperative relationship of DNMT-interacting proteins in DNAme and transcriptional regulation is an active field of study, especially with regards to DNMT1 activity.

DNAme profiles are also edited by TET enzymes, which convert 5-methylcytosine (5mC) to 5-hydroxymethylcytosine (5hmC) and subsequently induce DNA demethylation in a replication-dependent manner or via the base excision repair pathway [22–24]. Loss of TET activity predominantly affects induction of primitive endoderm (PrE) [25], primitive streak (PS) formation [26], and the removal of DNAme imprints in primordial germ cells (PGCs), which are the precursor cells of future germ cells, and in male meiosis [27–29]. *Dnmt3c*, a derivative of *Dnmt3b*, is not present in humans, but it is critical in retrotransposon methylation during mouse spermatogenesis [5]. Thus, all DNMT and TET enzymes play essential roles in regulating embryonic developmental processes and cell differentiation. However, neither *Dnmt1/3a/3b/3c* nor *Tet1/2/3* are required to maintain the undifferentiated cell state in mouse ESCs [30, 31]. Nevertheless, mouse ESCs have been used as an excellent tool for analyzing the function of these enzymes in epigenetic regulation during cell differentiation in culture because ESCs can be differentiated into epiblast-like cells (EpiLCs), which can then form functional somatic cells and PGC-like cells (PGC-LCs) *in vitro* [32]. Moreover, undifferentiated ESCs also provide a helpful tool for elucidating the cooperative function of these enzymes in epigenetic regulation.

In routine serum cell culture conditions, mouse ESCs represent an intermediate developmental stage between the inner cell mass cells and epiblasts. Their DNAme profile is dynamically regulated by the balance of opposing DNMT and TET activities, and at equilibrium, approximately 70% of the CpG sequences are methylated [33]. In recent years, high-throughput sequencing analysis such as whole-genome bisulfite sequencing (WGBS) has provided beneficial information about the distribution of either 5mC or 5hmC in coding regions. WGBS can reliably provide data on coding regions, intergenic regions, and ncRNA-coding genes. However, protein-coding genes comprise less than 3% of the human genome, and the rest of the genome contains highly repetitive, poorly fitting regions such as satellite, centromeric, and telomeric repeats [34]. Also, single-cell WGBS approaches have not yet been in general use, such that most analyses have been done on bulk cell populations. Therefore, most of the whole genome sequencing data for 5mC and 5hmC remain unutilized, especially in repeats that are preferential UHRF1–DNMT1 targets. Therefore, to comprehensively understand how, when, and to what extent each factor regulates genome-wide DNA modifications at the single-cell level, it will be essential to use other analysis methods in combination with WGBS.

Immunofluorescence (IF) staining using antibodies against DNA modifications, histone modifications, and histone variants is a common and reliable method for mapping specific epigenetic marks on animal and plant chromosomes [22]. In mammals, this method has often been used to distinguish active and inactive X chromosomes among the two X chromosomes present in every female somatic cell [35]. We previously performed IF staining for 5mC and 5hmC on mitotic chromosomes, which were prepared from wild-type (WT) ESCs, as well as *Dnmt3a*^*-/-*^ and *Dnmt3b*^*-/-*^ double knockout (DKO) ESCs. We found that DNMT3A/3B periodically remethylates unmodified double-stranded DNA (dsDNA) that appeared after the cell cycle-coupled removal of 5hmC. Conversely, by itself DNMT1 can maintain DNAme at pericentromeric heterochromatin regions, which escape DNA demethylation via TET [36, 37]. Therefore, IF staining has the advantage of analyzing dynamic changes in DNA modifications in each cell, providing helpful information for predicting when and where DNMT1/3s accessed dsDNA during cell cycles.

In this study, we performed IF staining of mitotic chromosomes that were prepared from mouse ESCs completely lacking the DNMT1 protein (1KO ESCs), and we found that DNMT1 may regulate the timing of DNAme by DNMT3s during the cell cycle. We also used DNMT1-deficient ESCs expressing a mutant DNMT1 without catalytic activity (DNMT1^CI^) and evaluated the catalytic activity-independent function of DNMT1 on gene expression and *de novo* DNAme during the early cell differentiation process from ESCs.

## Results

### DNA methylation defects visualized by antibody staining in *Dnmt1*^-/-^ mouse ESCs

**Fig 1:**
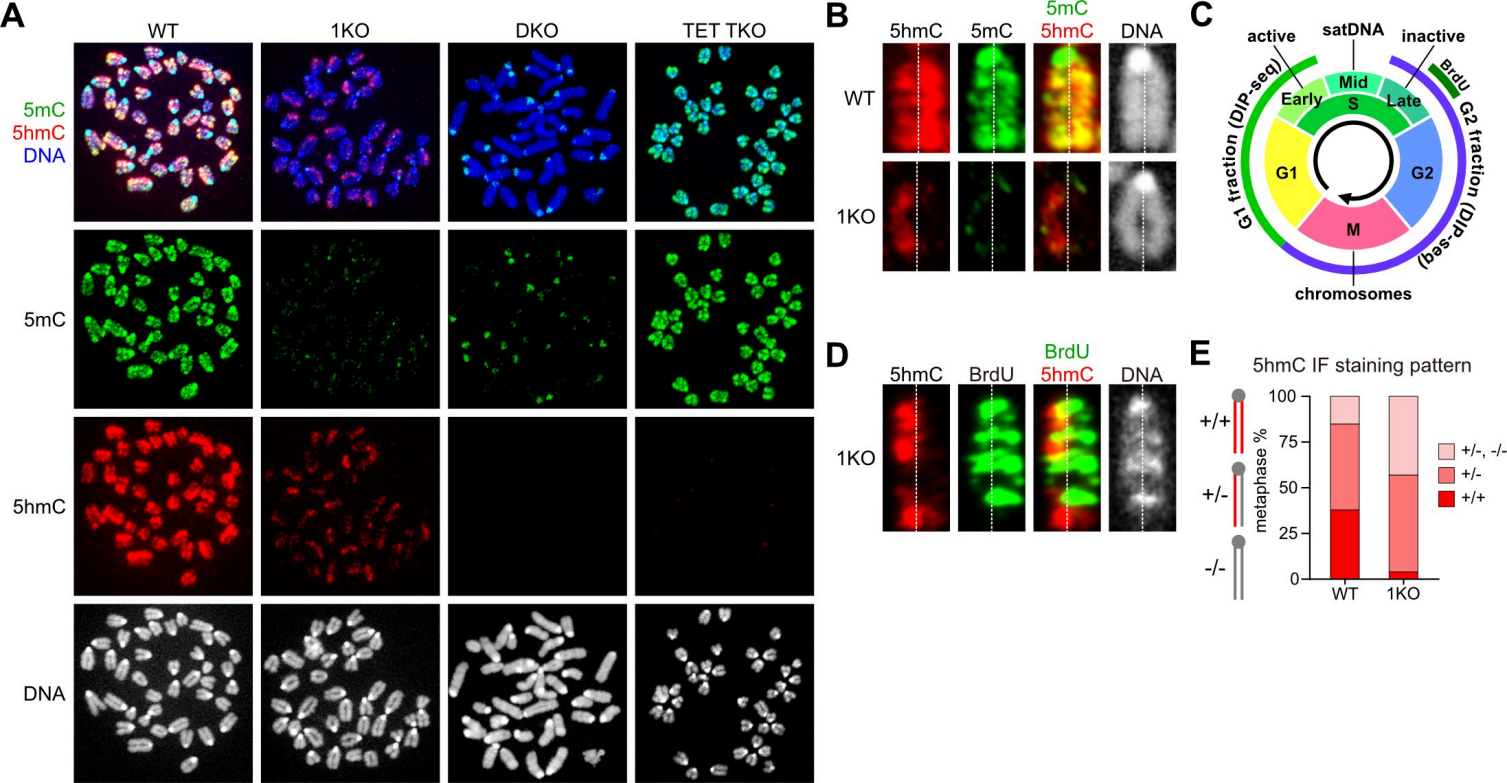
DNMT1 regulates *de novo* DNA methylation region and timing via DNMT3s. (**A**) Immunofluorescence (IF) staining patterns for 5-methylcytosine (5mC) and 5-hydroxymethylcytosine (5hmC) on metaphase chromosomes in DNMT1-deficient ESCs. WT, wild-type ESCs; 1KO, *Dnmt1*^-/-^ ESCs.; DKO, *Dnmt3a*^-/-^ and *Dnmt3b*^*-/-*^ ESCs; TET TKO, *Tet1*^-/-^, *Tet2*^-/-^, and *Tet3*^-/-^ ESCs. (**B**) Chromosomes show a typical IF staining pattern for 5mC and 5hmC in WT and 1KO ESCs. The dotted line indicates the space between two sister chromatids on a chromosome. (**C**) Schematic diagram showing the cell cycle information for the cell population that was used in this study. satDNA, pericentromeric heterochromatin rich in satellite repeats. (**D**) The late replicating region labeled with BrdU in S phase stains clearly with anti-BrdU antibody staining on both chromatids of each chromosome. (**E**) Mitotic cells were classified into the three categories: cells with most chromosomes that were equally positive for 5hmC on both sister chromatids (+/+); cells with the most chromosomes that were positive for 5hmC on one of the sister chromatids (+/−); cells that were mostly negative for 5hmC with some +/−chromosomes (+/−, −/−) (n = 66 WT and n = 51 1KO metaphases).

IF staining was first used to examine the overall differences in the distribution pattern of 5mC and 5hmC on metaphase chromosomes in wild-type (WT) ESCs, *Dnmt1* knockout (1KO) ESCs, *Dnmt3a/3b* double knockout (DKO) ESCs, and *Tet1/2/3* triple knockout (TET TKO) ESCs (Fig 1A). Two noteworthy IF staining features were observed in 1KO ESCs: (1) in many chromosomes, one of the sister chromatids is in an almost no 5hmC state; and (2) a significant reduction in 5mC IF signals in pericentric heterochromatin.

In 1KO ESCs, despite the presence of DNMT3 enzymes, the 5mC IF signals did not accumulate much in the heterochromatin around centromeres (Fig 1A). Conversely, DNMT1 preferentially maintains pericentromeric 5mC in DKO ESCs. Thus, the reduction of DNAme in pericentric heterochromatin may be mainly caused by the loss of maintenance DNMT activity or the lack of recruitment of DNMT3 to highly condensed heterochromatin where H3K9me2/3 is abundant, without the help of DNMT1. On the other hand, the absence of 5hmC IF signals on only one of the sister chromatids in the 1KO ESCs is not due to dysregulation of the DNMT3 target sequence recognition, because both sister chromatids on one chromosome are genetically identical (Fig 1B). Essentially, the TET enzyme can induce cell cycle-dependent DNA demethylation through a combination of the properties of 5hmC that prevent DNMT1 target recognition [38] and subsequent inhibition of *de novo* DNAme. Thus, chromatids composed mainly of unmodified dsDNA can be generated from hemi-hydroxymethylated dsDNA by transiently inhibiting *de novo* DNAme after DNA replication to metaphase.

There is a possibility that the difference in IF signal intensity of 5hmC between sister chromatids within each chromosome may be due to the difference in antibody-antigen accessibility that is caused by the low efficiency of DNA denaturation. To assess this possibility, cells were cultured in the presence of 5-bromodeoxyuridine (BrdU) 7 hours before cell preparation and used for chromosome preparation (Fig 1C). The chromosomal DNA was then denatured to expose the antigen to the antibody before IF staining was performed. Because BrdU was incorporated into the nascent DNA strand in the late S phase, the IF signal for BrdU appeared evenly on both sister chromatids. However, the IF signal for 5hmC was detected only on one of the sister chromatids (Fig 1D). Therefore, the 5hmC IF staining pattern seen in 1KO ESCs is not due to a technical artifact.

First, we focused on a previously undiscovered feature of 1KO ESCs, namely, that one of the sister chromatids is in an almost no 5hmC state (Fig 1B). The metaphases were classified into three categories based on the chromosome 5hmC staining pattern, as follows: (+/+), most chromosomes were equally positive for 5hmC on both sister chromatids; (+/−), most of the chromosomes that were positive for 5hmC on only one of the sister chromatids; and (+/−, −/−), some chromosomes were positive for 5hmC on only one of the sister chromatids and most chromosomes were negative for 5hmC. The total frequency of (+/−) and (+/−, −/−) cells in 1KO ESCs was 1.5-times higher than that in WT ESCs (Fig 1E, *p*<0.05).

These (+/−) and (+/−, −/−) 5hmC IF staining patterns are also observed in preimplantation mouse embryos. This is due to the broad conversion of paternally inherited 5mC to 5hmC by TET3 and subsequent dilution of 5hmC with DNA replication without *de novo* DNAme [22], suggesting that there is some defect in *de novo* DNAme in 1KO ESCs. In WT mouse ESCs, DNMT1 actively maintains pericentromeric DNAme in S phase, as observed in DKO ESCs (Fig 1A) [37]. In contrast, DNMT3s remethylates the newly synthesized unmodified dsDNA at any time throughout the cell cycle, and TET sequentially converts 5mC of euchromatin dsDNA to 5hmC. If *de novo* DNAme takes place only in G1 phase, the IF staining pattern of (+/−) 5hmC could be detected in most of metaphases without any complicated mechanism. This was explained using a model in the Discussion section. Thus, the IF staining pattern of (+/−) 5hmC detected in 1KO ESCs indicates that without DNMT1, DNMT3s are essentially incapable of methylating dsDNA, at least in the S and G2 phases (S/G2) before cells enter metaphase. However, DNMT3s actively methylate unmodified dsDNA mainly in the G1 phase (G1) before DNA replication, keeping the 5hmC level constant in 1KO ESCs (Fig 1A). Therefore, it is reasonable to speculate that DNMT3s methylate unmodified dsDNA during the S/G2 phase with the help of DNMT1 in WT ESCs.

### Overall reduction in 5hmC levels during the S phase of 1KO ESCs

**Fig 2:**
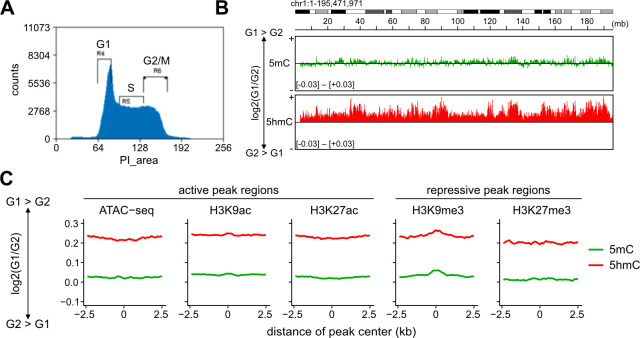
Restricted *de novo* DNA methylation after DNA replication in 1KO ESCs. (**A**) Cells were subdivided and collected using fluorescence-activated cell sorting (FACS) into the following two populations: either in G1 to early S phase (G1) or in late S to M via G2 phase (G2). Using MeDIP-seq and hMeDIP-seq, the distribution patterns of 5mC and 5hmC in the G1 and G2 populations were compared. (**B**) Differences in DNA modification patterns between G1 to G2. Chromosome 1 is shown as an example. The vertical axis takes a positive value when G1 is higher and a negative value when the G2 is higher. (**C**) Line plots showing the difference in 5mC and 5hmC between G1 and G2 of 1KO ESCs around ATAC-seq and histone modification peaks (±2.5 kb). Sequencing data from WT ESCs (GSE90895) was used to identify peaks for ATAC-seq and each type of histone modification.

Although the WGBS method can provide more accurate information on DNA modification levels, it cannot distinguish between 5mC and 5hmC. Therefore, we used methylated DNA and hydroxymethylated DNA immunoprecipitation sequencing (MeDIP-seq and hMeDIP-seq) to examine the dynamics of DNA modification through the cell cycle. First, we collected 1KO ESCs from G1 to early S phases (called G1) and 1KO ESCs in late S, G2, and M phases (called G2) using fluorescence-activated cell sorting (FACS) (Fig 2A). Then, two cell populations, G1 and G2, were used for Me/hMeDIP-seq. The results showed an overall decrease in 5mC and 5hmC in G2 cells (Fig 2B, chromosome 1 is an example that shows positive log2(G1/G2) values).

Next, we analyzed the distribution of 5mC and 5hmC in the transcriptionally active and inactive regions in two cell populations of 1KO ESCs, G1 and G2, respectively. The active regions were defined based on the peaks that were previously obtained from ATAC-seq and ChIP-seq analysis for H3K9ac and H3K27ac. In contrast, the inactive regions were determined based on those from ChIP-seq analysis for H3K9me3 and H3K27me3 enrichment in WT ESCs [39]. We then compared the distribution of both 5mC and 5hmC near the respective peaks between G1 and G2. While the 5mC values showed little change between G1 and G2, 5hmC values generally decreased in G2 compared with those in G1, regardless of active or inhibitory regions (as indicated by the positive values of log2(G1/G2) in Fig 2C). The decrease in the amount of 5hmC in G2 shown by Me/hMeDIP-seq in 1KO ESCs indicates that in the absence of DNMT1, DNMT3s do not remethylate unmodified dsDNA that appears after cell cycle-dependent 5hmC dilution throughout the S/G2 phase (see Fig 1C).

### Function of DNMT1 predicted from the phenotype of 1KO ESCs expressing a catalytically inactive mutant of DNMT1

**Fig 3:**
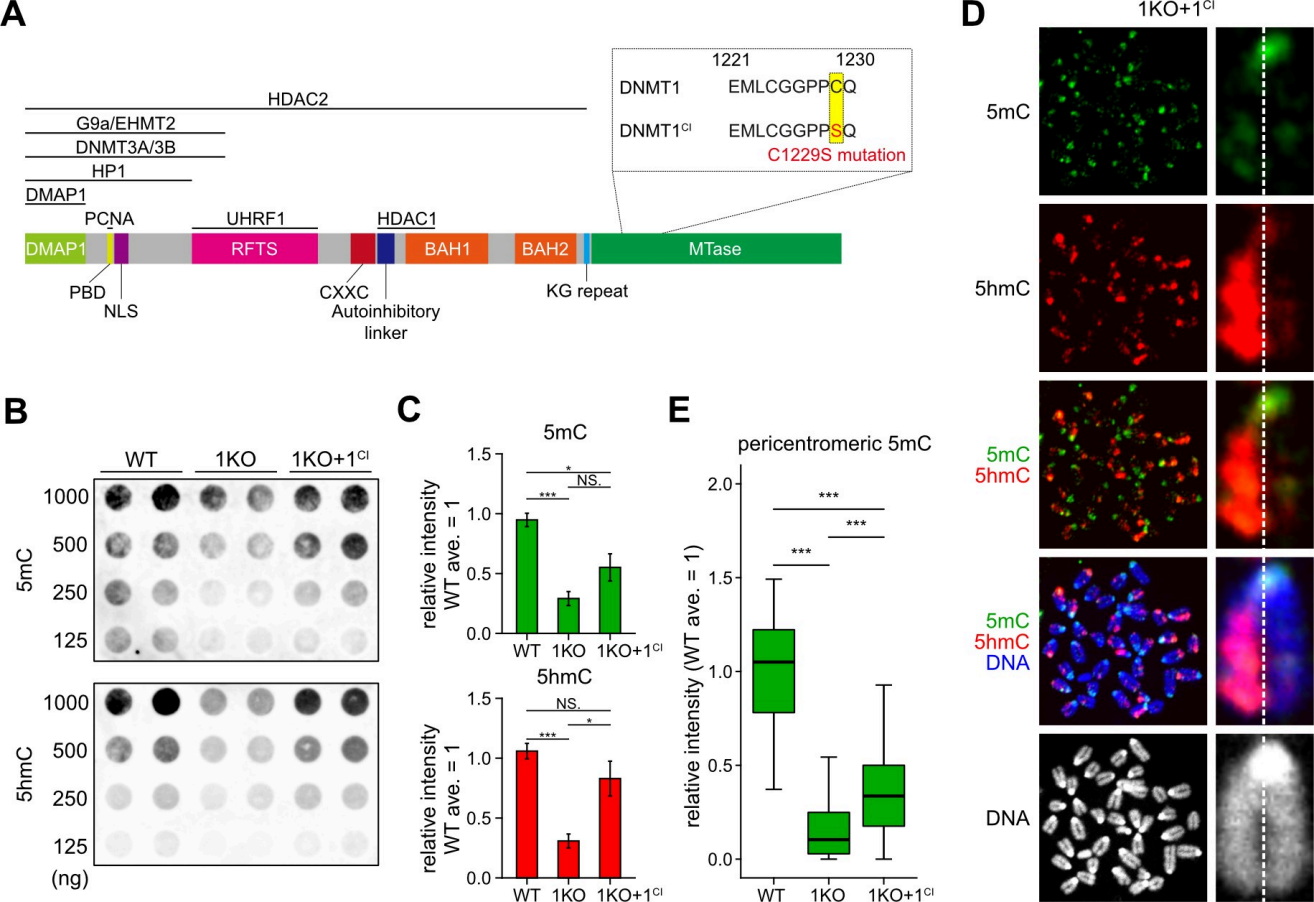
DNMT1^CI^, a catalytic null mutant of DNMT1, increases pericentric DNA methylation in 1KO ESCs. (**A**) DNMT1 domains and their interacting proteins. In DNMT1^CI^, the C1229S mutation is introduced in the catalytic domain by a single base substitution. (**B**) Dot blot analysis showing increased levels of 5mC and 5hmC in 1KO+1^CI^ ESCs compared to 1KO ESCs. (**C**) Quantification of the relative intensities of the 5mC and 5hmC signals obtained by dot blot analysis (n = 2; three blots). The average signal intensity obtained by WT ESCs was set to 1. (**D**) Chromosomes showing the typical IF staining patterns for 5mC and 5hmC in 1KO+1^CI^ ESCs. A dotted line indicates a gap of two sister chromatids in the chromosome. (**E**) Increased 5mC levels around centromeres in 1KO+1^CI^ ESCs detected by IF staining (***p<0.001).

DNMT1 interacts with a variety of proteins via its N-terminus [40, 41]. DNMT1 essentially requires UHRF1 as the N-terminal region binding proteins that recruit DNMT1 to hemimethylated dsDNA, replication foci, and heterochromatin for its function as a maintenance DNMT [17, 42]. In particular, the ubiquitination of the PCNA-associating factor PAF15 and histone H3 around heterochromatin rich in histone H3K9me3 by UHRF1 facilitates DNA binding of DNMT1 through the RFTS domain [43]. Therefore, since the interaction between DNMT1 and DNMT3s has been reported [44–46], DNMT1 may induce DNMT3s to access pericentromeric heterochromatin. To address this possibility, a DNMT1^CI^ expression vector was introduced into 1KO ESCs (Fig 3A). The transgene has a point mutation in exon 32 and encodes a C1229S mutant with no catalytic activity [47]. Transgenic 1KO ESCs expressing the transgene encoding DNMT1^CI^ under the CAG promoter (1KO+1^CI^ ESCs) were identified by genomic PCR between exons 1–2 using the primers listed in S1 Table. Here, two independent 1KO+1^CI^ ESC clones were established (S1A Fig) and analyzed in parallel, and representative images of one of them and the average of the two are shown as results. DNMT1^CI^ protein expression was confirmed by Western blot analysis (S1B Fig). Dot-blot analysis showed that both 5mC and 5hmC levels were increased in 1KO+1^CI^ ESCs compared to 1KO ESCs (Fig 3B and 3C). Since these cells do not contain any active DNMT1, our results suggest that DNMT1^CI^ promotes DNMT3-mediated DNAme in both heterochromatin and euchromatin.

Next, we examined the distribution patterns of 5mC and 5hmC in 1KO+1^CI^ ESCs using the IF method and found that DNMT1^CI^ did not rescue the 1KO ESC-specific IF staining pattern, namely one of the sister chromatids in the almost no 5hmC state (Fig 3D). Therefore, our results for 5hmC indicate that the catalytic activity of DNMT1 is required to induce DNMT3-mediated *de novo* methylation in the S phase. On the other hand, DNMT1^CI^ caused pericentromeric DNAme in 1KO+1^CI^ ESCs (Fig 3D and 3E), suggesting that DNMT1 probably interacts directly or indirectly with DNMT3s to facilitate the access of DNMT3s to dsDNA folded into heterochromatin.

### Expression of repetitive sequences in mouse ESCs without DNMT1

**Fig 4:**
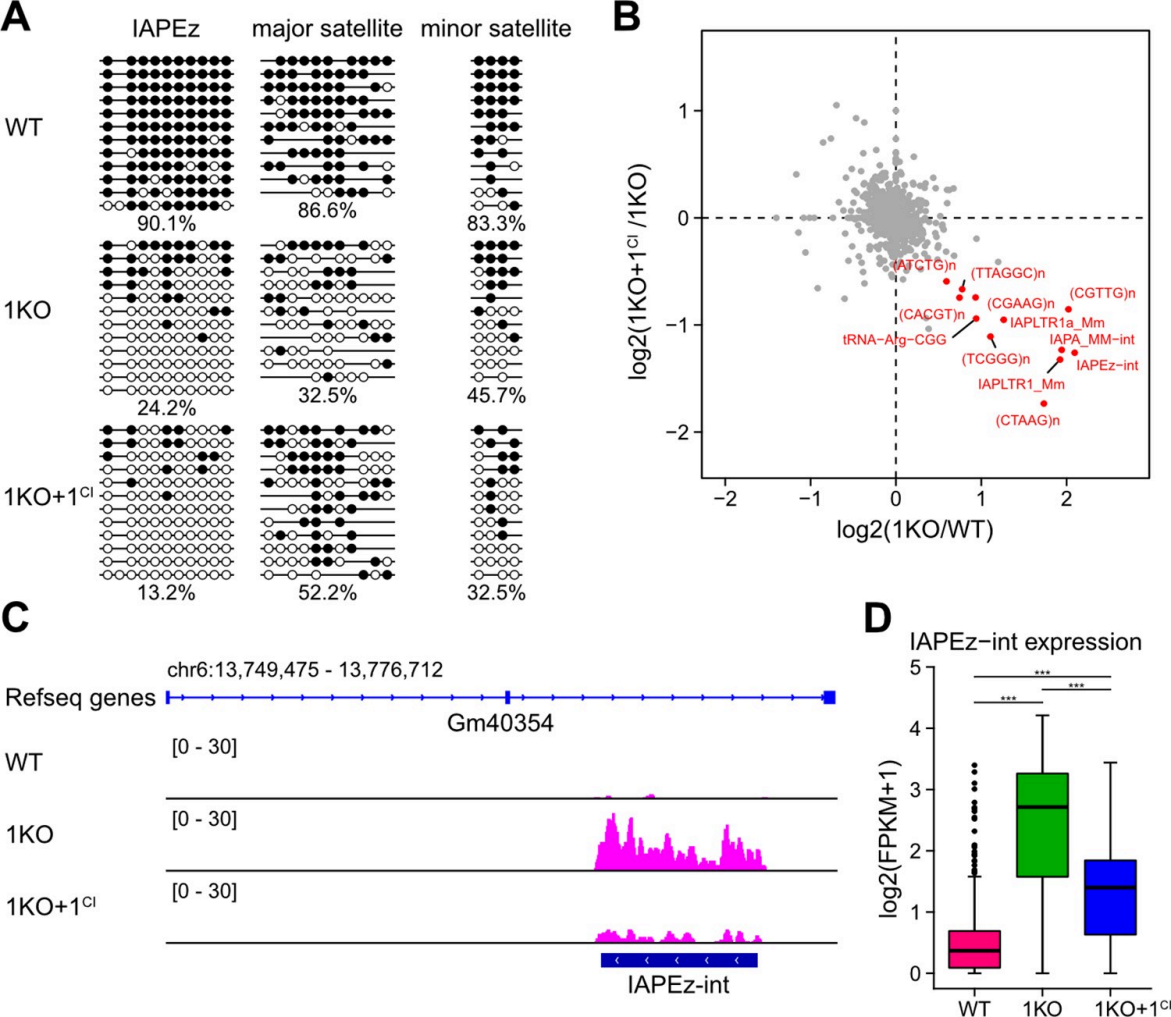
IAPEz-int repression without apparent acquisition of DNA methylation in 1KO+1^CI^ ESCs. (**A**) DNAme levels of repeat sequences detected by targeted bisulfite sequencing in ESCs. Black and white circles mean methylated and unmethylated CpG, respectively. (**B**) The scatter plot shows the repressive effect of DNMT1^CI^ on repetitive sequence expression in 1KO ESCs. Red dots, repeat sequence families, which are up-regulated in 1KO ESCs compared to WT ESCs (1KO / WT, log2 FC > 0.5), but down-regulated in 1KO+1^CI^ ESCs compared to 1KO ESCs (1KO+1^CI^ / 1KO, log2 FC < −0.5). (**C**) IAPEz-int expression located at Gm40354. A vertical axis, FPKM; a horizontal axis, corresponding genomic position. (**D**) IAPEz-int expression levels in WT, 1KO, and 1KO+1^CI^ ESCs (***p<0.001).

Mouse ESCs are often used as a model to study epigenetic gene regulation in pluripotent stem cells and during early embryonic development. In *Dnmt1* complete null mutant mice, repetitive sequences such as transposable elements and satellite DNA repeats are remarkably demethylated and highly expressed [13, 48, 49]. Because centromeric repeats were hypomethylated in 1KO ESCs but methylated in 1KO+1^CI^ ESCs as detected using IF staining, we investigated whether DNMT1^CI^ could lead to transcriptional repression from abundant repeats in ESCs using RNA-sequencing. Furthermore, using targeted bisulfite sequencing, we assessed the DNAme levels of IAPEz elements, one of the intracisternal A-particle (IAP) transposable elements, as well as major and minor satellite repeats (Fig 4A). DNMT1^CI^ did not alter DNA modification levels for IAPEz and minor satellite repeats in 1KO+1^CI^ ESCs (*p* = 0.31 and *p* = 0.34, respectively). However, the DNAme level at major satellite repeats was significantly increased in 1KO+1^CI^ ESCs compared to 1KO ESCs (*p* = 0.006). Thus, the accumulation of DNAme in repetitive sequences in WT ESCs can be attributed to DNMT1 maintaining DNAme, secondarily, to DNMT1 assisting with *de novo* DNAme by DNMT3s. We used RNA-seq to analyze the gene expression levels from several repetitive sequences in 1KO+1^CI^ ESCs (Fig 4B). In particular, transcription from IAPEz repeats was markedly repressed 1KO+1^CI^ ESCs (Fig 4C and 4D). Thus, DNMT1 may repress transcription from repetitive sequences in at least two ways: in a DNAme-dependent manner by recruiting DNMT3s and DNAme-independent manner by recruiting proteins that bind to DNMT1.

### Effect of DNMT1 on ESC differentiation independent of enzyme activity

**Fig 5:**
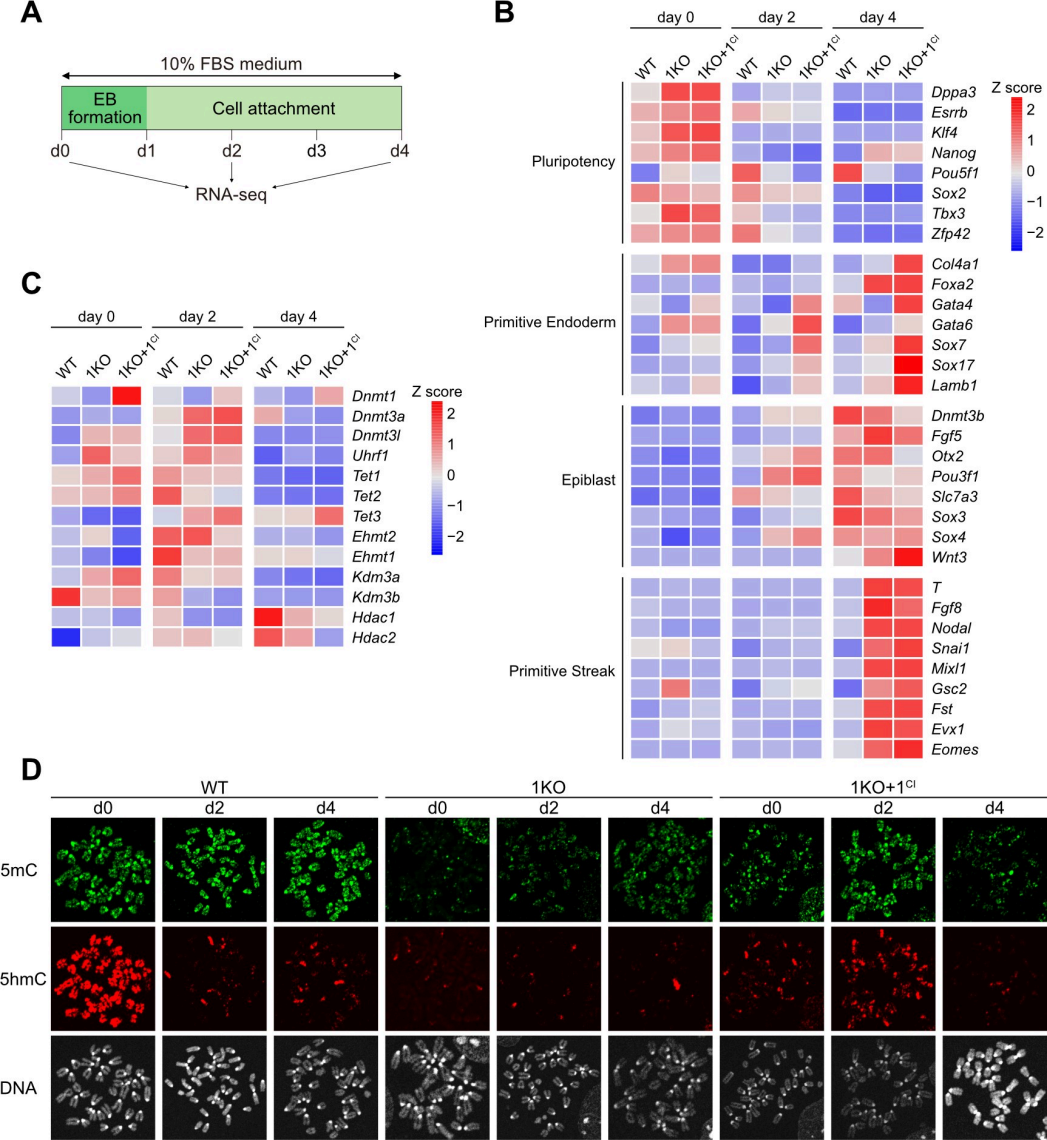
No positive effect of DNMT1^CI^ on the differentiation potency of 1KO ESCs. (**A**) The ESC differentiation procedure used here. Embryoid body (EB) formation initiates the differentiation of ESCs into epiblast-like cells (EpiLCs). Adherent cell culture of 1-day-old EBs was continued for 3 days to induce functional epiblast maturation. (**B**) Cell differentiation potency was compared between WT, 1KO, and 1KO+1^CI^ ESCs based on the gene expression profile detected by RNA-seq analysis. The heatmap shows Z-scores representing a relative difference in RNA expression levels in each gene between samples. 1KO+1^CI^ cells are more likely to differentiate into primitive endoderm-like cells rather than EpiLCs. (**C**) Expression profiles of genes related to epigenetic regulation. (**D**) IF staining patterns for 5mC and 5hmC on metaphase chromosomes showing global DNAme changes during 4-day cell differentiation in WT, 1KO, and 1KO+1^CI^ cells.

Next, we examined the effect of the methylase activity of DNMT1 on ESC differentiation. For this purpose, mouse ESCs were differentiated into EpiLCs via embryoid body (EB) formation in leukemia inhibitory factor (LIF)-free medium for 1 day. EBs were then cultured for another 3 days under adherent cell culture conditions to induce epiblast maturation (Fig 5A). Acquisition of high levels of DNAme through DNMT1, DNMT3A, and/or DNMT3B activity is essential for the differentiation of mouse epiblasts [50]. Therefore, DNMT1 and DNMT3s might co-regulate DNAme levels during epiblast formation. RNA-seq analysis was performed on day 0, day 2, and day 4 of differentiation for WT, 1KO, and 1KO+1^CI^ ESCs (Fig 5A). In day 4 WT cells, epiblast marker genes such as *Dnmt3b* and *Fgf5* were highly expressed. However, in 1KO and 1KO+1^CI^ cells, several epiblast marker genes were already expressed on day 2. In addition, in mouse embryogenesis, mesoderm and endoderm progenitors (called mesendoderm) are induced from epiblasts via primitive streak (PS) formation. In 1KO and 1KO+1^CI^ cells, *Wnt3* and *T/Brachyury*, which represent PS marker genes, were expressed on day 4 and showed faster differentiation characteristics than WT ESCs (Fig 5B). In 1KO cells, PrE marker genes were also abnormally expressed on day 4. Furthermore, in day 4 1KO+1^CI^ cells, the expression of PrE genes was even more markedly increased, and the expression of epiblast genes was limited (Fig 5B). RNA-seq confirmed *Dnmt1*^*CI*^ transgene expression during cell differentiation (Fig 5C). Remarkably, transcriptional repression of *Tet1* and *Tet2* was observed during EpiLC differentiation of the three cell lines analyzed.

IF staining of differentiated cells from 1KO and 1KO+1^CI^ ESCs compared to WT ESCs showed that during the differentiation of 1KO+1^CI^ ESCs, DNAme was increased genome-wide as well as WT ESCs by day 2 (Fig 5D), concomitant with transcriptional activation of *Dnmt3a/3b*. 5mC and 5hmC were diluted by day 4 with transcriptional repression of *Tet1* and *Tet2* during cell differentiation of 1KO+1^CI^ ESCs (Fig 5B and 5C). However, since the expression of *Tet1/2* is not so altered by day 2, chromatin-based inhibition of TET access may be dominantly responsible for the cell cycle-linked dilution of 5hmC in day 2 WT cells (Fig 5C and 5D). Thus, DNAme activity of DNMT1, accompanied by chromatin inactivation, may actively inhibit both PrE differentiation from ESCs and PS formation from EpiLCs in WT cells. In 1KO+1^CI^ cells, despite the overall increase in DNAme levels on day 2, PrE gene expression was increased. A possible explanation for this is a dominant-negative effect, whereby DNMT1^CI^ accumulates on the target of DNMT1 and may inhibit subsequent repression of PrE genes via DNMT3-catalyzed *de novo* DNA methylation.

## Discussion

**Fig 6:**
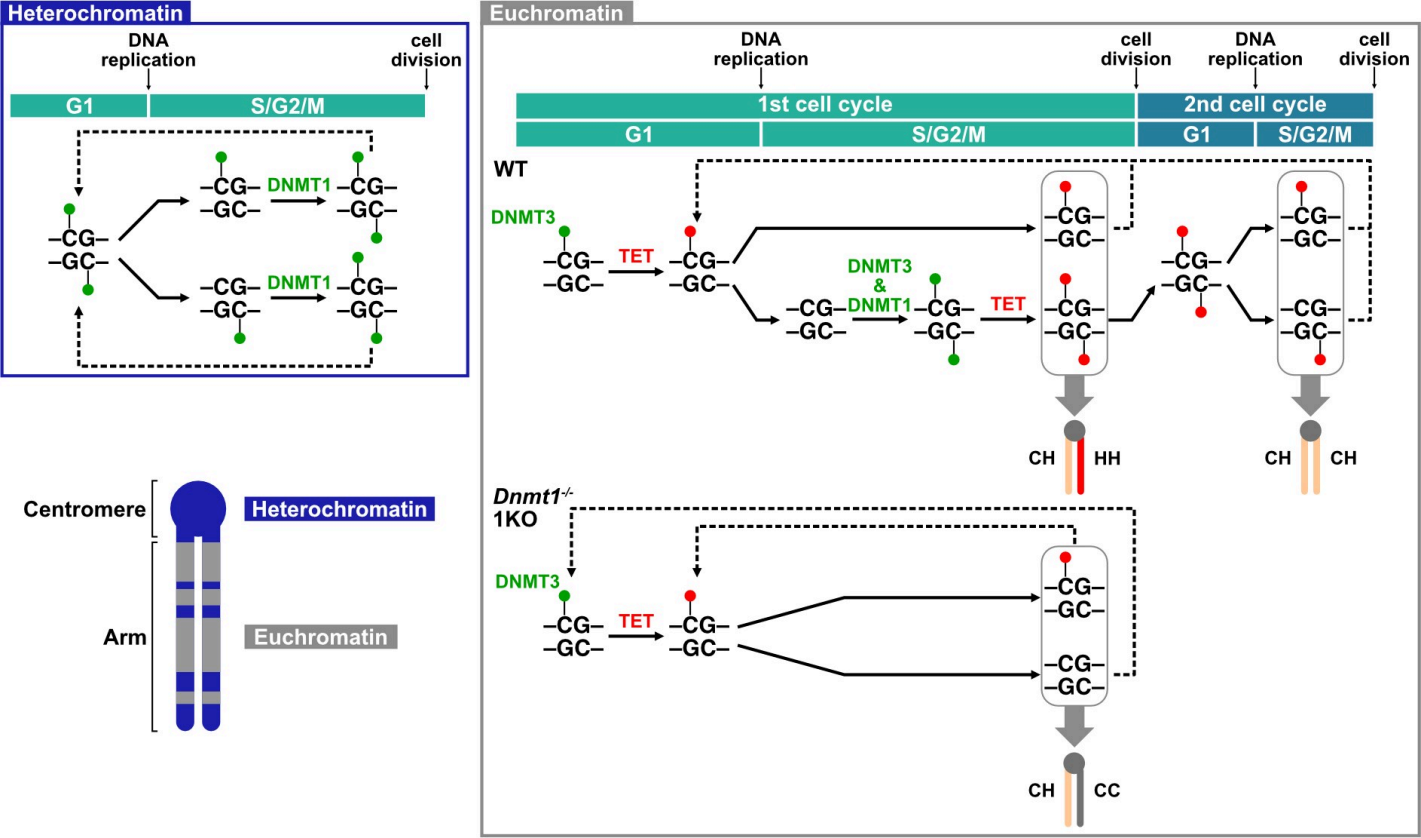
A model of DNA methylation regulation predicted from 5mC and 5hmC IF staining patterns in WT and 1KO ESCs. CH, hemi-hydroxymethylated double-stranded DNA (dsDNA); HH, fully hydroxymethylated dsDNA; CC, unmodified dsDNA.

In this study, IF staining analysis of metaphase chromosomes using anti-5mC and anti-5hmC antibodies revealed that, despite the presence of all enzymes in the DNMT3 family (DNMT3A/3B/3C/3L), ESCs lacking DNMT1 were unable to methylate newly synthesized dsDNA during the S–G2 phase. In 1KO+1^CI^ ESCs, DNMT1^CI^ with the catalytically inactive mutation at C1229S failed to induce detectable levels of DNAme by IF staining during the S–G2 phase. The results demonstrate that DNMT3 family enzymes are basically unable to methylate hemi-hydroxymethylated dsDNA throughout the cell cycle, and DNMT3s require DNMT1 enzymatic activity to remethylate unmodified newly synthesized dsDNA in euchromatin during the S phase (Fig 6). However, DNMT3s are solely able to remethylate unmodified DNA in the following G1 phase to recover a certain level of DNAme in euchromatin without DNMT1 (Fig 6, G1). Purified DNMT3A and 3B are able to methylate hemi-hydroxymethylated oligonucleotide substrates *in vitro* [51], but similar to DNMT1, DNMT3s have a very low tendency to methylate hemi-hydroxymethylated dsDNA in vitro [38, 52, 53]. Our results suggest that hemi-hydroxymethylated dsDNA is a poor substrate for DNMT3s *in vivo*, possibly via the action of TET enzymes or 5hmC readers [54]. For instance, the 5hmC reader UHRF2 was shown to interfere with DNMT3A function by promoting its ubiquitination-dependent degradation [55]. In contrast, what DNMT1 does during S phase in WT mouse ESCs is expected to recruit DNMT3s to the replication site and fully methylates DNMT3-mediated hemimethylated dsDNA by DNMT1’s maintenance methylation activity, while inhibiting TET function as DNMT1 occupies the replication site. Since *Dnmt1* KO ESCs induce the aggregation of heterochromatin through the accumulation of H3K27me3 and H2AK119ub by recruitment of PRC1 via TET1 [56], it can be reasonably assumed that TET’s access to DNMT1 targets is inhibited while DNMT1 is loaded in S phase (Fig 6, WT). Thus, in the G1 phase of DNMT1 knockout ESCs, when DNMT3-mediated DNAme is immediately converted to 5hmC by TET, the resulting hemi-hydroxymethylated dsDNA enters S phase without being fully methylated, and the unmodified double-stranded DNA generated by replication remains unmodified in the absence of DNMT1. This may explain why most chromosomes in *Dnmt1* knockout ESCs show the epigenetic asymmetry of sister chromatids (Fig 6, 1KO).

Contrary to this, DNMT1^CI^ was able to increase DNAme levels around centromeres of all chromosomes. DNMT1^CI^ also increased the genome-wide 5hmC level of 1KO ESCs. This may be due to increase *de novo* DNAme in the G1 phase, induced by DNMT1^CI^. This suggests that DNMT1^CI^ functions as an accessory factor for DNMT3s and might recruit DNMT3s to the DNMT1-target region through DNMT1–DNMT3 association, especially in the H3K9me-rich genomic regions. Thus, in wild-type ESCs, DNMT1 is proposed to regulate *de novo* DNAme together with DNMT3s in two ways: as an enzymatic activity-dependent function to induce DNAme by DNMT3 in the S phase and as an accessory factor to enhance the remethylation activity of DNMT3s, especially around pericentric heterochromatin in the G1 phase.

Interestingly, the expression of catalytically inactive DNMT1^CI^ protein leads to transcriptional repression of IAPEz, a family of long terminal repeat (LTR) retrotransposons, indicating in a DNAme-independent repression mechanism, and negatively affected the differentiation of EpiLCs by inducing more advanced PrE differentiation. Thus, although the catalytically inactive form of DNMT, such as DNMT1^CI^ protein, may sometimes have a dominant-negative effect on other relevant factors that regulate cell differentiation, this RNA-seq results basically suggest that the enzymatic activity of DNMT1 is important for the repression of PrE-specific genes.

In our previous IF staining in *Dnmt3a*^*-/-*^ and *Dnmt3b*^*-/-*^ DKO ESCs, we showed that DNMT3s cyclically remethylate the H3K4me2/3-rich euchromatic region of chromosomal arms after TET-mediated DNA demethylation. Similarly, heterochromatin around centromeres, enriched in H3K9me3, is continuously maintained by the maintenance DNMT1 activity over a long time, even in the absence of DNMT3s [36, 37]. DNMT3L may be involved in much of the DNAme of chromosomal arms as an accessory protein that directly binds to DNMT3A and DNMT3B and enhances their DNMT activity [6] because its loss induces a dramatic decrease in DNAme levels from 70% to 2% in *Dnmt3l*^*-/-*^ ESCs [33]. DNMT3L interacts with H3K36me3 and H3K36me2-rich regions and often binds to DNMT3A in oogenesis and spermatogenesis, respectively [5, 57–59]. Similarly, DNMT3B can associate with H3K36me2/3-rich regions in the gene bodies of actively transcribed genes in mouse ESCs [60–62]. These *de novo* methylations by DNMT3s are thought to be achieved during the G1 phase in 1KO ESCs. However, our IF staining results in 1KO, 1KO+1^CI^, and DKO ESCs highlights the phenomenon that the DNMT1 enzymatic activity is essential for *de novo* DNAme, possibly by DNMT3s that occurs in the S phase of WT ESCs.

There is no concrete evidence to discuss how the methylase activity of DNMT1 is involved in the DNMT3-mediated re-methylation in the S phase of WT ESCs. However, we are considering the following possibility. It was reported that DNMT1 fails to methylate hemimethylated site once every 20 hemimethylated sites, and sometimes introduce *de novo* methylation under in vitro system [19]. Furthermore, in recent studies, *de novo* methylation activity of DNMT1 has also been reported in vivo [20, 21, 30, 63]. Even if this *de novo* methylation activity is relatively low, the hemimethylated state created by DNMT1 may serve as an anchor for further accumulation of UHRF1–DNMT1. Subsequently, DNMT3 may be recruited directly or indirectly to the periphery via DNMT1, allowing the further spread of *de novo* DNAme on newly synthesized dsDNA *in cis* [46]. Subsequently, DNMT1 induces complete methylation of dsDNA by its maintenance of DNMT activity during the S–G2 phase. However, the involvement of another mechanism causing differences in 5hmC distribution between sister chromatids cannot be excluded.

As for the restoration of DNAme at pericentric heterochromatin in DNMT1^CI^, DNMT1 increases *de novo* DNAme by DNMT3s in an enzymatic activity-independent way. There are two possible ways for DNMT1 to enhance the methylase activity of DNMT3s: by directly binding to DNMT1 protein or through indirect association with DNMT1 via binding to a common interacting protein. The cooperative function of DNMT1 and DNMT3s in DNAme via direct interaction has been demonstrated [44–46]. Since 1KO ESCs showed less methylated in pericentromeric heterochromatin, direct interaction of DNMT3A/3B with histone methyltransferases (HMTase); H3K9me1/2 HMTases, G9a and SETDB1/ESET, an H3K9me3 HMTase, SUV39H1, and an H3K27me3 HMTase, EZH2 [64] may not be central to the DNAme accumulation in heterochromatin. The interaction of DNMT1 and DNMT3s may not be direct, as DNMT1 is reported to interact with many proteins such as PCNA and UHRF1 which can interact with the replication region of DNA, hemi-methylated dsDNA, and H3K9me2/3-positive heterochromatin [41, 65]. Additionally, the catalytic null mutant of G9a restored DNAme that was lost in *G9a*^-/-^ ESCs, which was unexpected [66]. G9a directly interacts both with DNMT3B/3A and DNMT1 within the PCNA complex [3]. DNMT1 can be recruited by UHRF1, which binds to a member of the PCNA complex, LIG1, which is itself methylated by G9a–GLP during DNA replication [67]. These reports suggest that the G9a protein may function as an intermediate protein that connects DNMT1 and DNMT3s during the S phase and increase DNAme levels in chromosomal arms. In addition, LSH/HELLS, an SNF-like ATPase helicase, interacts with both DNMT1 and DNMT3s, and its loss induces hypomethylation of DNA throughout the genome, including at repetitive sequences [68–70]. Furthermore, by interacting with LSH, DNMT1 represses gene expression in a catalytic activity-independent manner [71]. Therefore, we suggest LSH is another candidate for the repression of IAP elements and repetitive sequences in a DNAme independent manner. A simpler explanation is that the inhibition of TET access by DNMT1^CI^ may have indirectly increased the level of DNMT3-mediated DNAme. This is because TETs accumulate around centromeres in *Dnmt1*^-/-^ ESCs when DNMT1 is not present [56].

DNMT1 enzymatic activity is required for PrE gene repression [50, 72]. Briefly, in our ESC differentiation process, EB formation inhibits Wnt signaling to induce epiblast differentiation. Wnt/β-catenin signaling promotes PrE differentiation in the outer layer of cell aggregates formed primarily from ESCs [73]. The Nodal signaling pathway then induces mesendoderm formation through an event that mimics PS formation [74]. Thus, upregulation of PrE genes detected in 1KO and 1KO+1^CI^ cells demonstrated that the enzymatic activities of DNMT1 promote functional maturation of epiblasts by cooperating with DNMT3s to increase DNAme levels. High levels of DNAme may cause the nuclei of epiblasts to become unresponsive to extracellular signaling molecules (perhaps Wnt). This possibility is supported by the fact that human ESCs are in an early epiblast state and cannot maintain a pluripotent prime state without DNMT1, even in the presence of DNMT3A/3B [75].

The IF staining method used in this study is not ideal to accurately estimate the exact distribution and the amount of 5mC and 5hmC in the mouse genome. However, because the pair of sister chromatids are genetically identical in each chromosome, there is no alternative technique to detect epigenetic differences between sister chromatids. In recent studies, dsDNA from genetically different F1 hybrid cells and dsDNA marking only newly synthesized DNA strands with ethynyldeoxyuridine (EdU) or BrdU have been applied to WGBS analysis. However, these techniques can only analyze the epigenetic differences between the two paternal and maternal chromosomes and between the DNA strands in a dsDNA. Because WGBS could not distinguish between 5mC and 5hmC, we used hMeDIP-seq and demonstrated a global reduction of 5hmC in the G2 cell population compared to the G1 cell population. Therefore, the IF-mediated global DNA modification analysis is the only way to clarify the role of DNMT1 in actively regulating the timing and location of *de novo* DNAme by interacting with DNMT3s in ESCs and during cell differentiation. Our results provide important clues to elucidate the function of DNMT1 further.

## Materials and methods

### Cell lines and cultures

Mouse *Dnmt1*^-/-^ (1KO) ESCs were previously derived from J1 ESCs [11]. Transgenic ESC clones co-expressing a mutant gene encoding the catalytically inactive form of DNMT1 (DNMT1^CI^) and an IRES-linked zeocin resistance gene under the CAG promoter were generated by the lipofection method using the Lipofectamine™ LTX Reagent with PLUS™ Reagent (Thermo Fisher Scientific, Waltham, MA, USA). The transgenic cells were selectively grown in the ESC medium supplemented with 10 μg/mL of zeocin for 5 days. Thereafter, zeocin-resistant colonies were isolated and cultured individually in the ESC medium. DNA was isolated from each clone and genomic PCR was performed using the primers listed in S1 Table to identify the transgenic clones (S1 Fig and S1 File). The J1 ESCs were used as a wild-type (WT) control. All ESCs were cultured on mitomycin C-treated mouse embryonic fibroblasts (MEF) in ESC medium (D-MEM/Ham’s F12 [Wako, Osaka, Japan], 10% fetal bovine serum [Corning, NY, USA], 1× penicillin-streptomycin and 2 mM L-glutamate [Gibco^®^, Thermo Fisher Scientific, Waltham, MA, USA], 0.1 mM β-mercaptoethanol [Signa-Aldrich, St. Louis, MO, USA], and 10^3^ U/mL of ESGRO^®^ LIF [Merck Millipore, Burlington, MA, USA]) and grown under 5% CO_2_ at 37°C. The medium was changed daily.

### Cell differentiation

First, feeder cells were removed from the trypsinized ESCs by incubating for 30 min in a 100-mm gelatin-coated dish. The non-adherent ESCs were collected, and 2 × 10^7^ ESCs were placed in a 100-mm Petri dish. To form embryoid bodies (EBs), the cells were cultured in MEF medium (D-MEM supplemented with 10% FBS) for 1 day. One-day-old EBs were placed in a 6-well plate, and adherent cells were cultured in the MEF medium for 3 days. The medium was changed daily.

### Chromosome preparation

ESCs were treated with 0.3 μg/mL of colcemide (Demecolcine, Sigma-Aldrich) to enrich the mitotic cell population for 1 hour before harvesting. The trypsinized cells were treated with 0.075 M KCl at room temperature for 7 min and then gradually fixed by adding 1/10 volume of a Carnoy’s fixative solution (3:1 mixture of methanol and acetic acid) to the cell suspension. The cells were then fixed three times with 100% Carnoy’s fixative. Chromosomes were spread onto glass slides using an air-drying method.

### Immunofluorescence staining

Chromosomal DNA was denatured in 4N HCl at room temperature for 8 min and permeabilized with PBS-containing 0.1% Triton-X100 (Sigma Aldrich) for 10 min at room temperature. Samples were pre-blocked in 2% skim milk (Difco™, BD Biosciences, Franklin Lakes, NJ, USA)/PBS for 30 min at room temperature. Mouse monoclonal anti-5mC antibody (1:500) and rabbit polyclonal anti-5hmC antibody (1:500) (Active Motif, Carlsbad, CA, USA) diluted in 2% skim milk/PBS were used as primary antibodies. Anti-mouse IgG H&L Alexa Flour® 488 antibody (1:500) and anti-rabbit IgG H&L Alexa Flour® 546 antibody (1:500) (Invitrogen, Carlsbad, CA, USA) diluted in 2% skim milk/PBS were used as secondary antibodies. Antibody treatment was performed at room temperature for 1 hour. The sample was then washed three times in PBS containing 0.05% Tween 20 (PBST). Finally, the sample was mounted using Prolong™ Gold Antifade Mountant with DAPI (Invitrogen). Images of IF-stained specimens were captured with a Nikon A1 confocal microscope (Nikon, Tokyo, Japan).

### Dot blotting

Genomic DNA was sonicated to around 500 bp with Bioruptor^®^ (Cosmo Bio, Tokyo, Japan) and heat-denatured at 100°C for 10 min. The DNA was then blotted onto Hybond™-N^+^ nylon membranes (GE Healthcare) using Bio-Dot Microfiltration Apparatus (Bio-Rad Laboratories) and crosslinked with UV at 7 × 10^4^ μJ/cm^2^. The membrane was pre-blocked overnight at 4°C with 3% skim milk in PBS. The primary antibodies used here were the same as those used for IF staining. Treatment with each antibody diluted in 3% skim milk/PBS (1:1000) was performed at room temperature for 1 hour. The membrane was washed three times with PBST at room temperature and then treated with a secondary antibody, horseradish peroxidase (HRP)-conjugated anti-rabbit or anti-mouse IgG antibodies (GE Healthcare, Chicago, IL, USA) (1:1000) diluted in 3% skim milk/PBS at room temperature for 1 hour. Signals from dot blots detected with Clarity Max™ Western ECL Substrate (Bio-Rad Laboratories, Irvine, CA, USA) were acquired with Amersham Imager 680 (Cytiva, Tokyo Japan).

### Bisulfite sequencing

Sonicated and heat-denatured genomic DNA was applied to bisulfite conversion using the EZ DNA Methylation-Gold™ (Zymo Research, Irvine, CA, USA). The DNA was amplified with KOD -Multi & Epi-^®^ (TAKARA BIO, Shiga, Japan) using the primer sets using the primers listed in S1 Table. This PCR product was amplified once with Takara Ex Taq^®^ (TAKARA BIO, Shiga, Japan). The amplified DNA was cloned into TArget Clone™ (Toyobo, Osaka, Japan). Sequencing results were analyzed using a quantification tool for DNAme analysis, QUMA [76].

### RNA-seq

Total RNA was extracted using the RNeasy Plus Mini Kit (Qiagen, Hilden, Germany). Gene Nex RNA-seq analysis was performed using 1.6 μg of total RNA per sample extracted from undifferentiated mouse ESCs and differentiated cells from ESCs (Novogene, Beijing, China). Next Generation Sequencing was performed using Illumina’s NovaSeq 6000, PE150 system.

### Processing and analysis of RNA-seq data

Raw sequence reads from whole messenger RNA sequencing were trimmed to remove low-quality reads and adapter sequences using Trim Galore (https://github.com/FelixKrueger/TrimGalore) and then mapped to the mm10 genome using HISAT2 [77]. The Sam file was converted to a bam file using the SAMtools [78]. To visualize the bam file, a bigWig file was generated using the bamCoverage command from deepTools [79]. Gene expression and transposon expression were counted and normalized using analyzeRepeats.pl in HOMER software [80]. Integrative Genomics Viewer was used for visualizing bigWig files [81].

### Cell sorting

Feeder cell-free ESCs suspended in PBS were analyzed based on DNA content and immediately fractionated into three cell cycle phases using a FACSAria flow cytometer (BD Biosciences): a mixture of G1 and early-S (called G1), mid-S, and a mix of late-S, G2, and M (called G2). Next, genomic DNA was isolated from G1 and G2 fractions.

### MeDIP-seq and hMeDIP-seq

The MeDIP and hMeDIP libraries were generated according to the BC Cancer Agency Genome Sciences Centre protocol. One μg of genomic DNA sheared to 200–500 bp using the LE220 Focused-ultrasonicator (Covaris, Woburn, MA, USA) was end-repaired and A-tailed. The Illumina PE adapter-ligated DNA was heat-denatured and applied to MeDIP overnight with a monoclonal antibody for 5mC (AnaSpec, Fremont, CA, USA). hMeDIP was performed using a rat monoclonal antibody (Diagenode, Denville, NJ, USA) for 5hmC. The precipitated material was recovered with a secondary antibody of rabbit and rat anti-mouse IgG, respectively (Jackson ImmunoResearch, West Grove, PA, USA) along with protein A/G (Thermo Fisher Scientific), followed by digestion with 20 mg/mL Proteinase K (Invitrogen) for 2 hours. IP’ed DNA was purified using the MinElute PCR Purification Kit (Qiagen, Hilden, Germany). Confirmation of concentration of 5mC and 5hmC DNA was evaluated by qPCR using positive and negative control primers. The final library amplification was performed using Phusion® hot start II DNA polymerase and 5× Phusion® GC Buffer (Thermo Fisher Scientific) with Illumina paired-end index primers. The PCR-amplified library was size-selected for 220–450-bp fragments on an 8% PAGE gel. The library was normalized based on molarity and pooled for sequencing using v4 ready-to-load reagents for HiSeq 2500 System (Illumina, San Diego, CA, USA) at 75-bp paired-end reads.

### Processing and analysis of ChIP-seq and DIP-seq data

Published data were downloaded from GEO and converted from sra format to fastq format using a fastq-dump from the SRA Toolkit (http://ncbi.github.io/sra-tools/). Raw sequence reads were trimmed using Trim Galore, then mapped using Bowtie2 to mm10 genome [82]. Sam files were converted to Bam files using SAM tools. Peak calling for Assay for Transposase-Accessible Chromatin with high-throughput sequencing (ATAC-seq) and chromatin immunoprecipitation-sequencing (ChIP-seq) data were acquired with MACS2 [83]. DIP-seq data were normalized and generated wig files using MEDIPS [84]. Wig files were then converted to bigWig files using WigToBigWig (UCSC). Meta-plot was generated using the computeMatrix command from deepTools. Integrative Genomics Viewer was used for visualizing bigWig files.

### Statistical analysis

All statistical analyses were performed using R Statistical Software, version 4.1.0 (https://www.R-project.org/). Significance was determined using equal-variance Z values on both sides. Values of *p* < 0.05 were considered to be significant (*, *p*<0.05; **, *p*<0.01; ***, *p*<0.001).

## Supporting information

S1 Table PCR primer sets used in this study. (PDF)

S2 Table Datasets that were analyzed in this study. (PDF)

S1 Fig Isolation of transgenic *Dnmt1* complete-null mutant ESC clones, i.e. 1KO+1^CI^ ESCs expressing DNMT1^CI^, a catalytic-deficient mutant of DNMT1. (**A**) Selection of *Dnmt1* knockout (1KO) ESC clones bearing the *Dnmt1*^*CI*^ transgene by genomic PCR. PCR products of 918 bp and 122 bp were amplified from the endogenous *Dnmt1* allele and the transgene in 1KO+1^CI^ ESCs, respectively. (**B**) Western blot for DNMT1^CI^ protein levels in the 1KO+1^CI^ ESC clone. ACTB, loading control; WT and 1KO ESCs, positive and negative controls, respectively. Experimental methods to obtain the data shown in S1 Fig are described in the S1 File.
(PDF)

S1 File Experimental procedures related to the S1 Fig. (PDF)

S1 Raw images (PDF)
