# Supplementary material for: DNMT1 regulates the timing of DNA methylation by DNMT3 in an enzymatic activity-dependent manner in mouse embryonic stem cells
Source: PLoS One. 2022 Jan 5;17(1):e0262277. doi: 10.1371/journal.pone.0262277 (PMC8730390; doi:10.1371/journal.pone.0262277)
Supplement: S1 Table — (PDF) [file pone.0262277.s001.pdf]

S1 Table. PCR primer sets used in this study.

**Set**	**Primer**	**Sequence 5′–3′**
1	DNMT1 Ex1F	CAAATAGATCCCCAAGATCCAG
	DNMT1 Ex2R	CTCGTCCAAGTGAGTTTCCG
2	IAP Gag Fwd	ATTTTGTTGATTAAATAAATTATTATTGGG
	IAP Gag Rev	TAAAACATATCCTCTAATCATTTCTACTCA
3	Major satellite Fwd	GGAATATGGTAAGAAAATTGAAAATTATGG
	Major satellite Rev	CCATATTCCAAATCCTTCAATATACATTTC
4	Minor satellite Fwd	TAGAATATATTAGATGAGTGAGTTATATTG
	Minor satellite Rev	ATTATAACTCATTAATATACACTATTCTAC
