# Supplementary material for: DNMT1 regulates the timing of DNA methylation by DNMT3 in an enzymatic activity-dependent manner in mouse embryonic stem cells
Source: PLoS One. 2022 Jan 5;17(1):e0262277. doi: 10.1371/journal.pone.0262277 (PMC8730390; doi:10.1371/journal.pone.0262277)
Supplement: S2 Table — (PDF) [file pone.0262277.s002.pdf]

S2 Table. Datasets analyzed in this study.

Target	Cells	Type	GEO accession	Reference
5hmC	1KO-G1	DIP-seq	GSM5419624	This study
5hmC	1KO-G2	DIP-seq	GSM5419625	This study
5mC	1KO-G1	DIP-seq	GSM5419626	This study
5mC	1KO-G2	DIP-seq	GSM5419627	This study
open chromatin	WT	ATAC-seq	GSM2417076	Chronis et al., 2017
H3K9ac	WT	N-ChIP-seq	GSM2417092	Chronis et al., 2017
H3K27ac	WT	N-ChIP-seq	GSM2417096	Chronis et al., 2017
H3K27me3	WT	N-ChIP-seq	GSM2417100	Chronis et al., 2017
H3K9me3	WT	N-ChIP-seq	GSM2417112	Chronis et al., 2017
RNA	WT d0	RNA-seq	GSM5403455	This study
RNA	WT d2	RNA-seq	GSM5403456	This study
RNA	WT d4	RNA-seq	GSM5403457	This study
RNA	1KO d0	RNA-seq	GSM5403458	This study
RNA	1KO d2	RNA-seq	GSM5403459	This study
RNA	1KO d4	RNA-seq	GSM5403460	This study
RNA	1KO+1^{CI} d0	RNA-seq	GSM5403461	This study
RNA	1KO+1^{CI} d2	RNA-seq	GSM5403462	This study
RNA	1KO+1^{CI} d4	RNA-seq	GSM5403463	This study
