# Supplementary material for: DNMT1 regulates the timing of DNA methylation by DNMT3 in an enzymatic activity-dependent manner in mouse embryonic stem cells
Source: PLoS One. 2022 Jan 5;17(1):e0262277. doi: 10.1371/journal.pone.0262277 (PMC8730390; doi:10.1371/journal.pone.0262277)
Supplement: S1 Fig — (A) Selection of Dnmt1 knockout (1KO) ESC clones bearing the Dnmt1CI transgene by genomic PCR. PCR products of 918 bp and 122 bp were amplified from the endogenous Dnmt1 allele and the transgene in 1KO+1CI ESCs, respectively. (B) Western blot for DNMT1CI protein levels in the 1KO+1CI ESC clone. ACTB, loading control; WT and 1KO ESCs, positive and negative controls, respectively. Experimental methods to obtain the data shown in S1 Fig are described in the S1 File. (PDF) [file pone.0262277.s003.pdf]

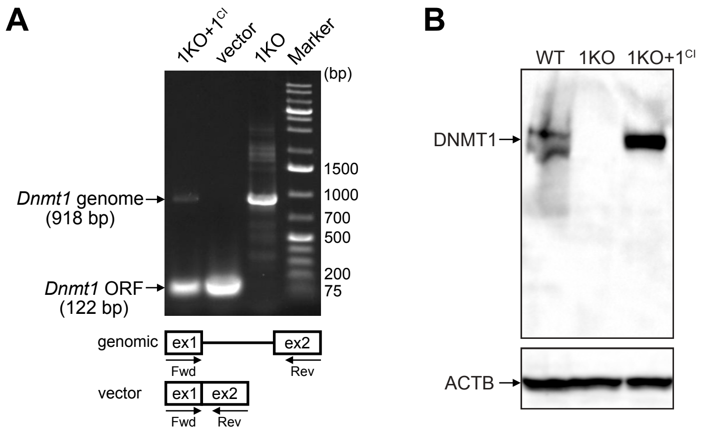

S1 Fig. Isolation of Dnmt1 complete-null nutant ESC clones (1KO+1CI ESCs), expressing DNMT1CI, a catalytic-deficient mutant of DNMT1.

(**A**) Selection of *Dnmt1* knockout (1KO) ESC clones bearing the *Dnmt1CI* transgene by genomic PCR. 918 bp and 122 bp PCR products were amplified from the endogenous *Dnmt1* allele and the transgene in 1KO+1CI ESCs, respectively. (**B**) Western blot for DNMT1CI protein levels in the 1KO+1CI ESC clone. ACTB, loading control; WT and 1KO ESCs, positive and negative controls, respectively. Experimental methods to obtain the data shown in **S1 Fig** are described in **S1 File**.
