# Supplementary material for: DNMT1 regulates the timing of DNA methylation by DNMT3 in an enzymatic activity-dependent manner in mouse embryonic stem cells
Source: PLoS One. 2022 Jan 5;17(1):e0262277. doi: 10.1371/journal.pone.0262277 (PMC8730390; doi:10.1371/journal.pone.0262277)
Supplement: S1 File — (PDF) [file pone.0262277.s004.pdf]

S1 File

Experimental procedures related to the S1 Fig.

Genomic PCR

Genomic DNA was isolated from cultured cells using standard procedures. The DNA was then denatured at 95˚C for 5 minutes and then subsequently amplified for 30 cycles (denaturation: 95˚C, 30 seconds, annealing: 55˚C, 30 seconds, and extension: 72˚C, 5 min) using ExTaq polymerase (TaKaRa, Shiga, Japan). The primers used for genomic PCR are shown in S1 Table to identify the transgenic clones.

Western blotting

After washing twice in 1 mM PMSF/PBS, cell lysates were extracted from cultured cells using RIPA buffer. Proteins were separated using e-PAGEL (ATTO, Tokyo, Japan) and transferred to the PVDF membrane (Merck Millipore, Burlington, MA, USA). The membrane was pre-blocked overnight at 4°C with 3% skim milk/PBS and then treated with the anti-DNMT1 antibody that was previously generated and evaluated in many studies [1] or anti-ACTB antibody (Abcam, Cambridge, UK) (1:500) diluted with 3% skim milk/PBS at room temperature for 1 hour. The membrane was washed three times with PBST at room temperature and then treated with a secondary antibody, horseradish peroxidase (HRP)-conjugated anti-rabbit or anti-mouse IgG antibodies (GE Healthcare, Chicago, IL, USA) (1:1000) diluted in 3% skim milk/PBS at room temperature for 1 hour. After washing three times with PBST, signals from Western blots detected with Clarity Max™ Western ECL Substrate (Bio-Rad Laboratories, Irvine, CA, USA) were acquired with Amersham Imager 680 (Cytiva, Tokyo Japan).

Reference

1. Garvilles RG, Hasegawa T, Kimura H, Sharif J, Muto M, Koseki H, et al. Dual functions of the RFTS domain of dnmt1 in replication-coupled DNA methylation and in protection of the genome from aberrant methylation. PLoS One. 2015;10: 1–19.
