# Supplementary figures and images for: DNMT1 regulates the timing of DNA methylation by DNMT3 in an enzymatic activity-dependent manner in mouse embryonic stem cells
Source: PLoS One. 2022 Jan 5;17(1):e0262277. doi: 10.1371/journal.pone.0262277 (PMC8730390; doi:10.1371/journal.pone.0262277)

Fig 3B

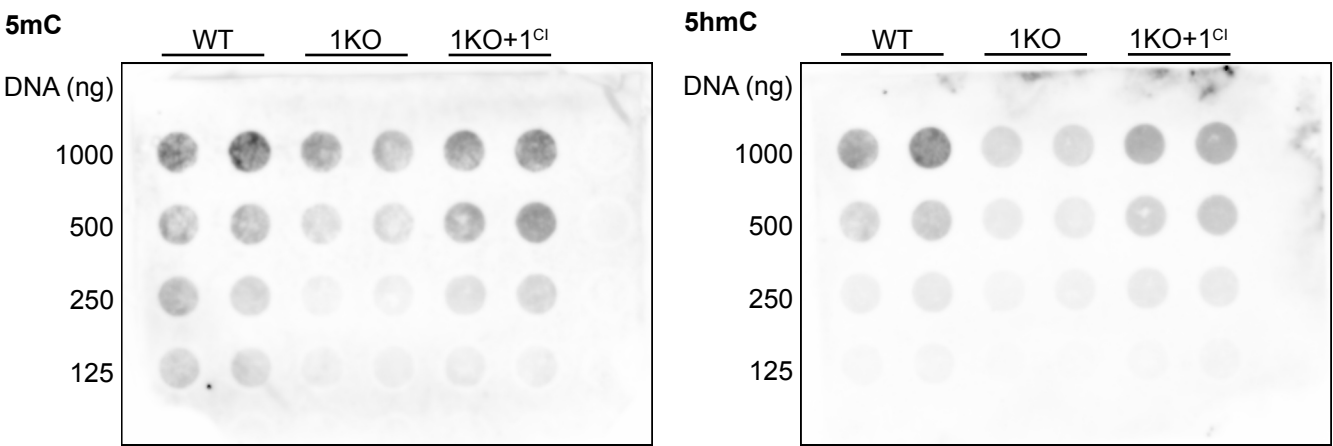

S1A Figure

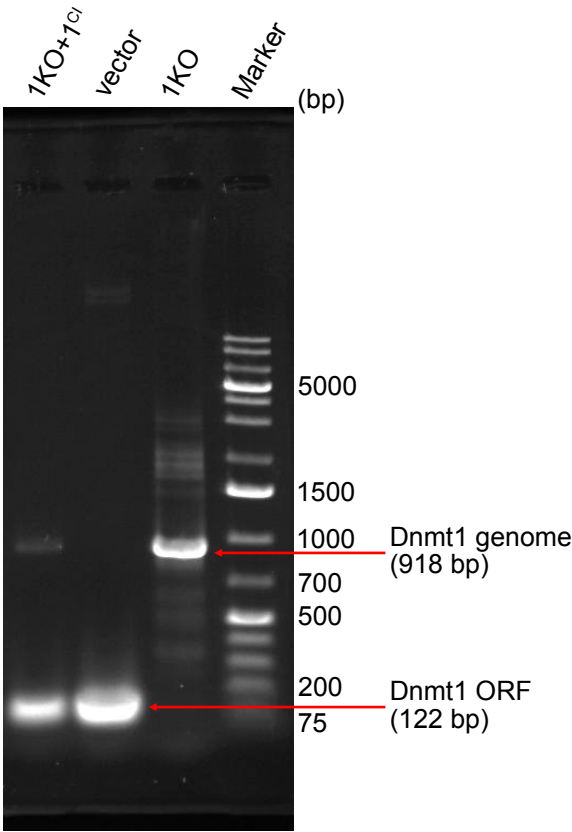

S1B Figure

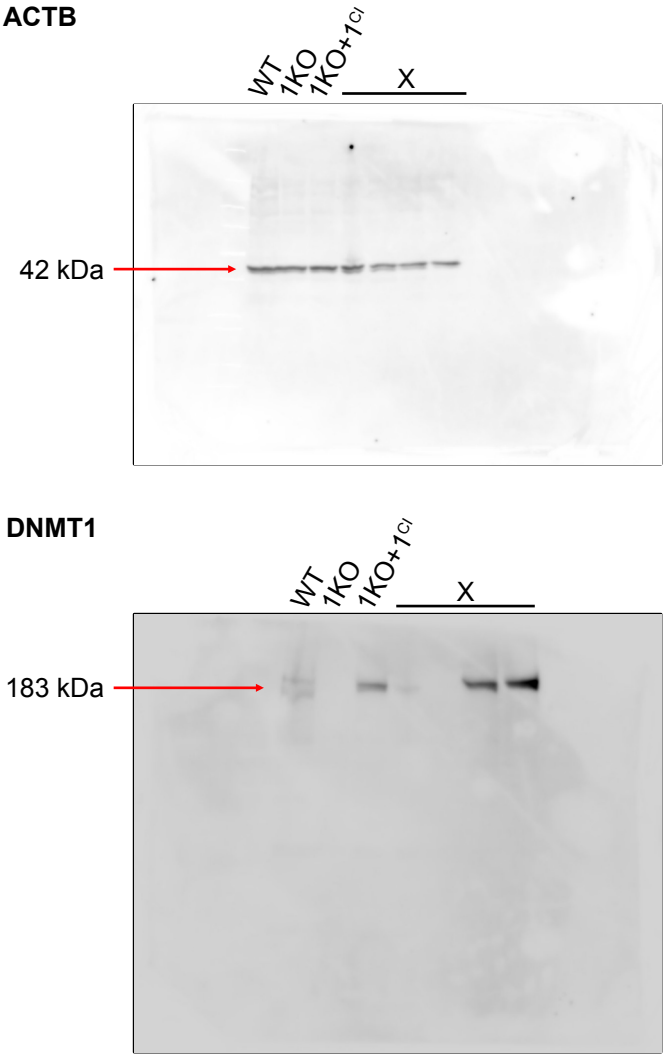

Supplement: S1 Raw images — (PDF) [file pone.0262277.s005.pdf]
